# Protecting maternal health: Strategies against HIV and malaria in pregnancy

**DOI:** 10.1097/MD.0000000000039565

**Published:** 2024-09-06

**Authors:** Emmanuel Ifeanyi Obeagu, Getrude Uzoma Obeagu

**Affiliations:** a Department of Medical Laboratory Science, Kampala International University, Ishaka, Uganda; b School of Nursing Science, Kampala International University, Ishaka, Uganda.

**Keywords:** antiretroviral therapy, HIV, malaria, maternal health, pregnancy, preventive measures

## Abstract

Maternal health remains a global priority, with particular emphasis on combating infectious diseases such as HIV and malaria during pregnancy. Despite significant progress in prevention and treatment efforts, both HIV and malaria continue to pose significant risks to maternal and fetal well-being, particularly in resource-limited settings. The prevention of mother-to-child transmission (PMTCT) programs for HIV and intermittent preventive treatment (IPTp) for malaria represent cornerstone strategies in mitigating the impact of these infections on pregnancy outcomes. PMTCT programs focus on early HIV diagnosis, antiretroviral therapy initiation, and promoting safe infant feeding practices to reduce the risk of mother-to-child transmission. Similarly, IPTp involves the administration of antimalarial medication to pregnant women in malaria-endemic regions to prevent maternal and fetal complications associated with malaria infection. Integration of HIV and malaria prevention and treatment services within existing maternal and child health programs is crucial for maximizing impact and minimizing healthcare system strain. Strengthening health systems, improving access to antenatal care services, and enhancing community engagement are essential components of comprehensive maternal health strategies. Furthermore, promoting awareness, education, and empowerment of pregnant women and communities are vital in fostering health-seeking behaviors and adherence to preventive measures against HIV and malaria. In conclusion, protecting maternal health from the dual threat of HIV and malaria requires a multifaceted approach that encompasses prevention, screening, treatment, and community engagement.

## 1. Introduction

Maternal health remains a cornerstone of global public health initiatives, striving to ensure the well-being of both the mother and child throughout the delicate phase of pregnancy.^[1]^ However, the coexistence of Human Immunodeficiency Virus (HIV) and Malaria in pregnant women poses a formidable challenge, significantly impacting maternal health outcomes worldwide.^[2]^

HIV, a viral infection with profound immunological implications, and Malaria, a mosquito-borne parasitic disease, independently jeopardize the health of pregnant women.^[3]^ Yet, when they converge within the same individual, their combined impact amplifies, posing intricate clinical and public health challenges.

Maternal health remains a critical concern globally, particularly in regions where infectious diseases such as HIV and malaria pose significant threats to pregnant women and their unborn children. The intersection of pregnancy with these diseases presents unique challenges, necessitating effective strategies for prevention, management, and treatment. HIV and malaria infections during pregnancy not only jeopardize the health of mothers but also increase the risk of adverse outcomes such as preterm birth, low birth weight, and mother-to-child transmission of infections. Consequently, there is a pressing need to explore and implement comprehensive approaches to safeguard maternal health and ensure positive birth outcomes in areas affected by these diseases. The complexity of addressing HIV and malaria in pregnancy demands a multifaceted approach that encompasses both preventive measures and clinical interventions. Prevention strategies, including antiretroviral therapy (ART) for HIV and intermittent preventive treatment (IPTp) for malaria, have demonstrated efficacy in reducing the incidence and impact of these diseases on maternal and fetal health. However, the effectiveness of these interventions may vary depending on factors such as geographical location, healthcare infrastructure, and socio-economic determinants. Understanding the landscape of available interventions and their respective strengths and limitations is crucial for informing policy decisions and guiding healthcare practices aimed at protecting maternal health. Moreover, advancements in biomedical research and public health initiatives have led to the development and implementation of innovative interventions targeting HIV and malaria in pregnancy. These may include novel drug regimens, vaccine development, community-based interventions, and integration of maternal health services within existing healthcare systems. By harnessing a combination of biomedical, behavioral, and structural interventions, it is possible to address the multifaceted nature of maternal health challenges posed by HIV and malaria.^[4,5]^

This paper aims to comprehensively analyze the intertwined complexities of HIV and Malaria co-infection during pregnancy, shedding light on the epidemiology, pathophysiology, clinical considerations, and strategies devised to safeguard maternal health.

## 2. Aim

The aim of this study is to examine the effectiveness and impact of strategies aimed at protecting maternal health against HIV and malaria during pregnancy. Specifically, the study seeks to evaluate the implementation of prevention of mother-to-child transmission (PMTCT) programs for HIV and IPTp for malaria in pregnant women. Additionally, the study aims to assess the integration of HIV and malaria prevention and treatment services within existing maternal and child health programs, with a focus on healthcare system strengthening and community engagement. Through this research, we aim to contribute to evidence-based approaches for enhancing maternal health outcomes and reducing the burden of HIV and malaria infections on pregnant women and their infants.

## 3. Rationale

Maternal health is a critical public health concern, particularly in regions where HIV and malaria infections pose significant risks to pregnant women and their offspring. Understanding the rationale behind addressing these dual burdens is imperative for designing effective interventions and policies aimed at safeguarding maternal health. HIV and malaria are major contributors to maternal morbidity and mortality globally, especially in resource-limited settings. Pregnant women are particularly vulnerable to these infections due to immunological changes during pregnancy, increasing their susceptibility to severe complications. Both HIV and malaria infections during pregnancy can have adverse effects on maternal health, leading to complications such as anemia, low birth weight, preterm birth, and mother-to-child transmission. Addressing these infections is crucial for improving pregnancy outcomes and reducing maternal and neonatal mortality rates. Prevention strategies such as PMTCT programs for HIV and IPTp for malaria have demonstrated effectiveness in reducing the transmission and impact of these infections on pregnant women and their infants. Understanding the rationale behind these interventions is essential for promoting their widespread implementation and ensuring optimal coverage and adherence among pregnant women. Integrating HIV and malaria prevention and treatment services within existing maternal and child health programs maximizes resources, improves efficiency, and enhances access to comprehensive care for pregnant women. This integrated approach addresses the overlapping vulnerabilities and ensures a holistic response to maternal health challenges. Strengthening health systems to deliver integrated maternal health services is essential for sustainability and long-term impact. By building capacity, improving infrastructure, and enhancing health workforce skills, health systems can better meet the needs of pregnant women affected by HIV and malaria.

## 4. Review methodology

### 4.1. Search strategy

**Database selection:** A systematic search was conducted across multiple electronic databases, including PubMed, MEDLINE, Embase, Scopus, and the Cochrane Library. These databases were selected for their extensive coverage of biomedical literature and relevant studies in the field of maternal health and infectious diseases.

**Search terms:** A comprehensive set of search terms was developed to ensure the inclusion of relevant studies. These terms included variations and combinations of keywords such as “maternal health,” “pregnancy,” “HIV,” “malaria,” “interventions,” “prevention,” and “treatment.” Boolean operators (AND, OR) were used to refine search queries and maximize search results.

**Inclusion criteria:** Studies were included if they met the following criteria:

○ Published in peer-reviewed journals.
○ Written in English.
○ Focused on interventions or strategies for preventing or managing HIV and malaria in pregnant women.
○ Reported outcomes related to maternal health or birth outcomes.

**Exclusion criteria:** Studies were excluded if they:

○ Were conducted exclusively in non-human subjects.
○ Did not focus on pregnant women.
○ Did not report relevant outcomes related to maternal health or birth outcomes.
○ Were conference abstracts, editorials, or commentaries without original data.

**Publication date:** No restrictions were imposed on publication dates to ensure the inclusion of both recent and older studies, providing a comprehensive overview of the literature.

#### 4.1.1. Study selection process

**Screening:** Titles and abstracts of retrieved studies were independently screened by 2 reviewers to assess their relevance based on the inclusion and exclusion criteria outlined above.

**Full-text review:** Full-text articles of potentially relevant studies were retrieved and independently assessed by 2 reviewers for eligibility and relevance.

**Data extraction:** Data from included studies were extracted using a standardized form, capturing information on study characteristics (e.g., author(s), year of publication, study design), participant characteristics, interventions or strategies evaluated, outcomes measured, and key findings.

**Quality assessment:** The methodological quality of included studies was evaluated using appropriate tools such as the Cochrane Collaboration’s tool for assessing risk of bias in randomized trials or the Newcastle-Ottawa Scale for observational studies.

## 5. Epidemiology of HIV and malaria in pregnant women

The epidemiology of HIV and Malaria in pregnant women is a critical aspect in understanding the prevalence, distribution, and impact of these co-infections on maternal health outcomes.^[6]^ According to the World Health Organization (WHO), millions of pregnant women globally are affected by HIV, particularly in sub-Saharan Africa, which bears a substantial burden.^[7]^ Without intervention, there’s a risk of mother-to-child transmission of HIV during pregnancy, childbirth, or breastfeeding.^[8]^ Effective ART has significantly reduced transmission rates.^[9]^ HIV prevalence among pregnant women varies geographically, influenced by factors such as access to healthcare, education, socioeconomic status, and cultural practices.^[9]^ HIV poses significant health risks to pregnant women, affecting their immune system and increasing susceptibility to opportunistic infections. Co-infection with Malaria exacerbates health complications.^[10]^

Malaria, caused by Plasmodium parasites transmitted through the bite of infected mosquitoes, predominantly affects pregnant women in sub-Saharan Africa, Southeast Asia, and parts of Latin America.^[11]^ Pregnant women are particularly susceptible to Malaria due to changes in immunity during pregnancy, making them more prone to severe complications, including anemia, low birth weight, and maternal mortality. Strategies such as the use of insecticide-treated bed nets, IPTp, and prompt diagnosis and treatment are pivotal in reducing Malaria-associated risks in pregnant women.^[12]^ Malaria in pregnancy contributes significantly to adverse outcomes, including maternal anemia, miscarriage, stillbirth, preterm delivery, and low birth weight, thereby impacting both maternal and fetal health.^[13]^ When HIV and Malaria coexist in pregnant women, their combined impact worsens health outcomes, increasing the risks of complications for both the mother and the unborn child.^[14]^ Regions with high HIV prevalence often coincide with areas endemic for Malaria, leading to a higher likelihood of co-infection, which further complicates clinical management and treatment.^[15]^ Efforts integrating HIV and Malaria prevention and treatment services aim to address these co-infections efficiently, emphasizing the need for comprehensive healthcare services tailored to the specific needs of pregnant women.^[16]^

## 6. Pathophysiology and clinical considerations

During pregnancy, childbirth, or breastfeeding, there’s a risk of mother-to-child transmission of HIV.^[17]^ This can occur when the virus crosses the placenta, during delivery, or through breast milk. Pregnancy induces alterations in the maternal immune system to tolerate the fetus, which might impact the progression of HIV. Changes in cellular immunity can affect the control of viral replication. HIV compromises the immune system, increasing vulnerability to opportunistic infections and complicating pregnancy-related conditions.^[18]^ ART aims to suppress viral load and mitigate these risks. Pregnant women, particularly primigravidae, are at higher risk due to reduced immunity against Plasmodium falciparum-infected red blood cells that sequester in the placenta.^[19]^ Infected red blood cells adhere to placental tissue, causing inflammation, impairing placental function, and leading to complications like low birth weight, preterm delivery, and maternal anemia.^[20]^ Malaria in pregnancy increases the risk of adverse outcomes for both the mother and fetus, contributing to maternal anemia, stillbirths, miscarriages, and intrauterine growth restriction.^[13]^

The pathophysiology of HIV and malaria during pregnancy involves intricate mechanisms that impact maternal health and fetal outcomes. In the case of HIV, the virus targets immune cells, particularly CD4 + T lymphocytes, leading to systemic immune suppression. During pregnancy, physiological changes, such as alterations in hormonal levels and immune modulation, can exacerbate HIV infection, increasing the risk of disease progression and vertical transmission to the fetus. Additionally, the placenta serves as a potential reservoir for HIV, facilitating transmission to the developing fetus through various mechanisms, including transplacental passage or exposure during delivery. Malaria, caused by Plasmodium parasites transmitted through the bite of infected mosquitoes, presents distinct challenges in pregnancy. Pregnant women are more susceptible to malaria infection due to changes in immunity and alterations in the structure and function of the placenta. Plasmodium falciparum, the most virulent species, can sequester in the placenta, leading to placental malaria, which is associated with adverse outcomes such as maternal anemia, low birth weight, and increased risk of perinatal mortality. The interplay between maternal immunity, placental factors, and parasite virulence influences the severity of malaria infection and its impact on maternal and fetal health.^[13,18–20]^

## 7. Clinical considerations in HIV and malaria co-infections

Co-infection exacerbates the pathophysiological consequences of both HIV and Malaria. HIV may worsen Malaria infection severity, leading to increased placental damage and adverse outcomes.^[21]^ Identifying co-infections requires comprehensive testing protocols. However, in resource-limited settings, access to accurate diagnostic tools for both infections can be limited. Managing both HIV and Malaria concurrently in pregnant women demands a careful balance of antiretroviral drugs and anti-malarial treatments to prevent drug interactions and ensure optimal outcomes for both infections.^[22]^ Integrated antenatal care programs that combine screening, prevention, and treatment strategies for both HIV and Malaria aim to improve maternal and fetal health outcomes. In clinical practice, managing HIV and malaria in pregnancy requires a comprehensive approach that addresses both maternal and fetal health needs. For HIV, ART plays a central role in preventing mother-to-child transmission (PMTCT) by suppressing viral replication and reducing the viral load in maternal blood and genital secretions. Initiating ART early in pregnancy and maintaining adherence throughout gestation are crucial for optimizing maternal health outcomes and reducing the risk of vertical transmission. In the case of malaria, preventive strategies such as IPTp with antimalarial medications, insecticide-treated bed nets, and vector control measures are recommended to reduce the risk of infection and its complications. Early diagnosis and prompt treatment of malaria episodes during pregnancy are essential to prevent severe maternal outcomes and mitigate the adverse effects on fetal development. Close monitoring of maternal parasitemia, hemoglobin levels, and fetal growth is warranted to identify and manage complications promptly. Furthermore, the management of HIV and malaria in pregnancy requires interdisciplinary collaboration among obstetricians, infectious disease specialists, pediatricians, and allied healthcare professionals. Integrating antenatal care, PMTCT services, and malaria prevention into routine maternal health programs can enhance access to comprehensive care and improve maternal and child health outcomes. Additionally, addressing social determinants of health, such as poverty, inadequate healthcare infrastructure, and stigma, is crucial for mitigating barriers to care and promoting maternal well-being in resource-limited settings.^[21,22]^

## 8. Strategies for prevention and control

Initiating ART early in pregnancy for HIV-positive women significantly reduces the risk of vertical transmission.^[23]^ Consistent adherence to ART during pregnancy and breastfeeding is crucial. Maintaining viral suppression through ART not only benefits the mother’s health but also reduces the risk of mother-to-child transmission of HIV.^[24]^ Administering preventive anti-malarial drugs (such as sulfadoxine-pyrimethamine) at specified intervals during pregnancy reduces the risk of Malaria infection and related complications. Distribution and promotion of Insecticide-Treated Nets (ITNs) to pregnant women help reduce mosquito bites, lowering the risk of Malaria infection.^[25]^ Integrating HIV and Malaria prevention, screening, and treatment services into routine Antenatal Care (ANC) visits ensures comprehensive care for pregnant women.^[26]^ Incorporating syndromic management approaches where feasible can aid in diagnosing and treating both infections simultaneously, especially in resource-limited settings with limited diagnostic facilities.

Implementing universal access to ART for pregnant women living with HIV is a cornerstone of prevention and control efforts. Early initiation of ART during pregnancy, ideally before conception or as soon as HIV infection is diagnosed, significantly reduces the risk of mother-to-child transmission (MTCT). Combination ART regimens, including nucleoside reverse transcriptase inhibitors (NRTIs), non-nucleoside reverse transcriptase inhibitors (NNRTIs), protease inhibitors (PIs), and integrase inhibitors, are recommended to achieve viral suppression and prevent vertical transmission. Prevention of Mother-to-Child Transmission (PMTCT) Programs aim to provide comprehensive services for preventing HIV transmission from mother to child throughout the antenatal, intrapartum, and postpartum periods. Key components of PMTCT include HIV testing and counseling, provision of ART for pregnant and breastfeeding women, infant prophylaxis with antiretroviral medications, safe delivery practices, and support for adherence to treatment and retention in care. IPTp for Malaria involves the administration of antimalarial medication, typically sulfadoxine-pyrimethamine, to pregnant women during antenatal care visits, regardless of malaria symptoms or parasitemia. IPTp aims to prevent malaria infection, reduce the burden of placental malaria, and mitigate adverse maternal and fetal outcomes. Regular IPTp dosing, usually starting in the second trimester and repeated at scheduled intervals, is recommended in malaria-endemic regions. ITNs and Vector Control provide physical barriers to prevent mosquito bites and reduce the risk of malaria transmission during sleep. Distributing ITNs to pregnant women and promoting their proper use, especially in endemic areas with high malaria transmission rates, is an effective strategy for malaria prevention. Additionally, indoor residual spraying (IRS) of insecticides and environmental management to reduce mosquito breeding sites contribute to vector control efforts. Routine screening for HIV and malaria during antenatal care visits allows for early detection and treatment of asymptomatic infections, thereby reducing the risk of complications and transmission to the fetus. Prompt diagnosis and management of HIV and malaria in pregnant women, coupled with adherence support and follow-up care, are essential components of integrated maternal health services. Health education and behavior change communication strategies play a vital role in promoting uptake of preventive measures and fostering positive health-seeking behaviors among pregnant women. Providing accurate information on HIV and malaria prevention, addressing misconceptions, and empowering women to make informed decisions about their health and that of their unborn child contribute to comprehensive prevention and control efforts. Integrating HIV and malaria prevention and control services within existing maternal and child health programs facilitates access to comprehensive care and improves health outcomes for pregnant women and their infants. Strengthening health systems, enhancing capacity for diagnosis and treatment, ensuring a reliable supply chain for essential medications and commodities, and promoting community engagement are critical for sustaining effective prevention and control initiatives.^[23–26]^

## 9. Health education and behavioral interventions

Educating pregnant women about the risks of HIV and Malaria co-infections, the importance of antenatal care, adherence to medication, and preventive measures like ITN usage.^[27]^ Involving local communities, community health workers, and peer educators in disseminating information, promoting health-seeking behaviors, and encouraging ANC attendance.^[28]^ Enhancing access to diagnostic tools, medications, and skilled healthcare professionals in areas with a high prevalence of HIV and Malaria.^[29]^ Addressing socioeconomic factors such as poverty, transportation challenges, and cultural beliefs that hinder access to healthcare services.^[30]^ Investing in research aimed at developing more effective preventive measures, treatment protocols, and interventions specifically tailored for pregnant women with HIV and Malaria co-infections.^[31]^ Regular surveillance to track the prevalence, trends, and effectiveness of implemented interventions, aiding in evidence-based decision-making and program adjustments. Implementing a multi-faceted approach that combines medical interventions, education, community involvement, and healthcare infrastructure improvements is essential to effectively prevent and control HIV and Malaria co-infections in pregnant women, thereby safeguarding maternal and fetal health.^[31]^

Providing comprehensive health education during antenatal care visits is essential for empowering pregnant women with knowledge about HIV and malaria prevention and management. Antenatal counseling sessions offer opportunities to discuss the importance of adherence to ART for HIV-positive women, the benefits of IPTp for malaria, and the proper use of ITNs. Tailoring educational messages to address cultural beliefs, misconceptions, and individual concerns enhances the effectiveness of health promotion efforts. Encouraging pregnant women to adopt healthy behaviors that reduce their risk of HIV and malaria infection is paramount. This includes promoting safer sexual practices, such as condom use and partner testing for HIV, to prevent sexual transmission of the virus. Similarly, advocating for the consistent use of ITNs, adherence to IPTp dosing schedules, and avoidance of mosquito bites through environmental modifications and personal protection measures are critical for malaria prevention. Engaging communities through targeted education campaigns raises awareness about HIV and malaria prevention and encourages community participation in health promotion activities. Community health workers, peer educators, and local leaders can serve as trusted sources of information and support, delivering culturally sensitive messages and facilitating discussions on prevention strategies, treatment options, and available support services. Behavior changes communication interventions utilize various communication channels, including mass media, social media, interpersonal communication, and community outreach, to promote positive health behaviors among pregnant women and their families. Creative approaches such as storytelling, role-playing, drama, and visual aids can effectively convey health messages, address misconceptions, and motivate behavior change within the community. Establishing peer support groups and group counseling sessions provides opportunities for pregnant women to share experiences, receive emotional support, and learn from each other’s coping strategies. Peer educators who have lived experience with HIV or malaria can serve as role models and sources of inspiration, promoting adherence to treatment, destigmatizing the diseases, and fostering a sense of solidarity within the community. Integrating health education and behavioral interventions into routine maternal and child health services optimizes access to information and support for pregnant women. This may involve incorporating health education sessions into antenatal care visits, linking pregnant women to community resources and support groups, and strengthening referral systems for additional counseling and psychosocial support as needed. Continuous monitoring and evaluation of health education and behavioral interventions are essential for assessing their impact, identifying areas for improvement, and ensuring accountability. Key performance indicators may include knowledge uptake, behavior change outcomes, retention in care, and satisfaction with services. Regular feedback from participants and stakeholders informs programmatic adjustments and enhances the effectiveness and sustainability of interventions over time.^[27–31]^

## 10. Challenges and barriers

Certainly, addressing HIV and Malaria co-infections in pregnant women is accompanied by various challenges and barriers that can impede prevention, diagnosis, and treatment efforts. Remote or rural areas often lack adequate healthcare infrastructure, making it challenging for pregnant women to access essential services and medications. Insufficient healthcare facilities, trained personnel, and diagnostic tools in certain regions hinder timely diagnosis and proper management of HIV and Malaria co-infections.^[32]^ Financial constraints limit access to medications, transportation to healthcare facilities, and consistent antenatal care visits, impacting adherence to treatment protocols.^[33]^ Lack of education and awareness about health issues can lead to misconceptions, delays in seeking care, or noncompliance with preventive measures.

Social stigma related to HIV/AIDS might prevent pregnant women from seeking HIV testing, disclosing their status, or adhering to treatment due to fear of discrimination or social ostracization.^[34]^ In resource-limited settings, the availability and accuracy of diagnostic tests for both HIV and Malaria might be limited, leading to challenges in accurate diagnosis and treatment.^[35]^ Managing co-infections requires careful consideration of potential drug interactions between antiretroviral drugs for HIV and anti-malarial medications. Adherence to multi-drug regimens for both HIV and Malaria can be challenging, especially for pregnant women who might experience side effects or difficulties in maintaining consistent medication schedules.^[36]^ Cultural beliefs, myths, and traditional practices may influence healthcare-seeking behaviors, affecting ANC attendance, medication adherence, and acceptance of preventive measures.^[37]^ Lack of integration between HIV and Malaria programs or vertical healthcare systems might hinder the coordination of services, leading to missed opportunities for comprehensive care.^[38]^

## 11. Maternal and fetal outcomes

Maternal and fetal outcomes in cases of HIV and Malaria co-infections during pregnancy can be significantly impacted by various factors. HIV and Malaria co-infection may accelerate disease progression in HIV-positive pregnant women, leading to increased susceptibility to opportunistic infections and other complications.^[14,27,39–42]^ Complications such as anemia, thrombocytopenia, and severe illness may arise due to the combined effects of both infections.^[43]^ Increased risk of adverse maternal outcomes including preterm delivery, low birth weight, stillbirth, and miscarriage due to the impact of both infections on maternal health. Severe cases of co-infections can lead to maternal mortality, especially in settings with limited access to healthcare or in cases of delayed diagnosis and treatment. Some medications used for treating HIV and Malaria co-infections may have side effects that can impact maternal health and require careful management.^[44–48]^ Untreated or poorly managed HIV and Malaria co-infections increase the risk of vertical transmission of both infections to the fetus, leading to congenital HIV infection and/or congenital Malaria.^[49]^ Co-infections increase the likelihood of delivering preterm or having infants with low birth weight, which are associated with increased risks of neonatal morbidity and mortality. Infants born to mothers with untreated co-infections are at higher risk of infectious diseases, developmental issues, and compromised immune systems, impacting their long-term health. Severe co-infections during pregnancy can contribute to neonatal mortality, particularly in cases of extremely premature births or in infants with complications related to co-infections.^[49]^

Maternal outcomes in pregnancies affected by HIV and malaria are influenced by various factors, including disease severity, access to healthcare, and adherence to preventive and therapeutic interventions. In the context of HIV, untreated infection during pregnancy can lead to increased maternal morbidity and mortality, including progression to acquired immunodeficiency syndrome (AIDS), opportunistic infections, and maternal death. However, effective ART significantly improves maternal health outcomes by suppressing viral replication, preserving immune function, and reducing the risk of HIV-related complications. One of the primary concerns in pregnancies affected by HIV and malaria is the risk of vertical transmission of infections from mother to child. Without intervention, HIV can be transmitted during pregnancy, childbirth, or breastfeeding, leading to perinatal HIV infection in the infant. Similarly, malaria parasites can cross the placenta and infect the fetus, resulting in adverse outcomes such as low birth weight, preterm birth, and stillbirth. Prevention strategies such as ART for HIV and IPTp for malaria are effective in reducing vertical transmission rates and improving neonatal health outcomes. Maternal HIV and malaria infections are associated with an increased risk of adverse birth outcomes that can impact both short-term and long-term health outcomes for the infant. Infants born to HIV-positive mothers may experience higher rates of preterm birth, low birth weight, and intrauterine growth restriction, as well as an increased risk of neonatal morbidity and mortality. Similarly, malaria in pregnancy is linked to complications such as placental insufficiency, fetal growth restriction, and congenital malaria, which can contribute to adverse birth outcomes and neonatal complications. The consequences of maternal HIV and malaria infections extend beyond the immediate perinatal period and can have long-term implications for child health and development. Children born to HIV-positive mothers may face challenges related to HIV exposure, including the need for early diagnosis, access to ART, and ongoing medical monitoring. Additionally, prenatal exposure to malaria may increase the risk of neurocognitive impairment, susceptibility to other infections, and impaired growth and development in childhood. Maternal mortality and morbidity rates are disproportionately higher among pregnant women affected by HIV and malaria, particularly in resource-limited settings with limited access to healthcare services. Complications such as severe anemia, malaria-related organ dysfunction, and HIV-associated opportunistic infections contribute to maternal mortality, highlighting the urgent need for comprehensive prevention and treatment strategies. Improving access to maternal healthcare services, strengthening health systems, and addressing social determinants of health are critical for reducing maternal morbidity and mortality associated with these infections. Integrated maternal health services that address the dual burden of HIV and malaria are essential for optimizing maternal and fetal outcomes. Coordinated efforts to provide antenatal care, PMTCT services, malaria prevention and treatment, and comprehensive obstetric care contribute to improved maternal health, reduced vertical transmission rates, and better birth outcomes. Multidisciplinary collaboration among healthcare providers, policymakers, and community stakeholders is crucial for implementing evidence-based interventions and addressing the complex challenges posed by HIV and malaria in pregnancy.^[43–49]^ Table 1 shows ART regimens for prevention of mother-to-child transmission (PMTCT) of HIV, Table 2 shows key components of integrated antenatal care for HIV and malaria prevention and Table 3 shows strategies to improve adherence to antiretroviral therapy (art) in pregnant women (provided by the authors).

**Table 1:** Antiretroviral therapy (ART) regimens for prevention of mother-to-child transmission (PMTCT) of HIV.

ART regimen	Components	Administration
Option A	AZT (zidovudine) during pregnancy, NVP (nevirapine) at onset of labor, and 7 days postpartum	Sequential
Option B	Triple ART regimen (usually 2 NRTIs + 1 NNRTI or PI) initiated during pregnancy and continued throughout breastfeeding	Concurrent
Option B+	Lifelong triple ART regimen (usually TDF/3TC/EFV) initiated during pregnancy regardless of CD4 count and continued indefinitely	Concurrent

**Table 2:** Key components of integrated antenatal care for HIV and malaria prevention.

Component	Activities
HIV Testing and Counseling	Voluntary counseling and testing (VCT), provider-initiated counseling and testing (PICT), opt-out approach
Malaria Screening	Rapid diagnostic tests (RDTs), microscopy, assessment of malaria symptoms
Intermittent Preventive Treatment (IPTp)	Administration of sulfadoxine-pyrimethamine (SP) during antenatal visits, usually starting in the second trimester
Insecticide-Treated Bed Nets (ITNs)	Distribution of ITNs, education on proper use and maintenance
Health Education	Information on HIV and malaria prevention, ART adherence, IPTp, and ITN usage

**Table 3:** Strategies to improve adherence to antiretroviral therapy (ART) in pregnant women.

Intervention	Description	Outcome
SMS reminders	Automated text messages reminding women to take their medications	Improved adherence rates
Peer support	Peer mentorship and support groups for women on ART	Enhanced retention in care
Community health workers	Home visits and counseling by trained community health workers	Increased treatment adherence

## 12. Future directions and recommendations

Promote further integration of HIV and Malaria prevention, diagnosis, and treatment services within existing maternal healthcare programs to provide comprehensive care during antenatal visits.^[50]^ Increase investment in healthcare infrastructure, diagnostic tools, medications, and skilled healthcare professionals in regions heavily burdened by HIV and Malaria co-infections.^[51]^ Provide training programs for healthcare workers to improve their capacity for managing co-infections, including handling drug interactions and offering comprehensive care. Encourage research for the development of innovative preventive measures, vaccines, and treatment strategies specifically tailored for pregnant women with HIV and Malaria co-infections. Invest in research to develop and implement accurate, cost-effective, and accessible diagnostic tools suitable for resource-limited settings to enhance early detection and treatment initiation.

Conduct community-based health education initiatives to raise awareness about the risks of co-infections, the importance of ANC visits, adherence to medication, and the use of preventive measures.^[52]^ Advocate for policies that support integrated maternal healthcare services and ensure equitable access to quality care for pregnant women affected by HIV and Malaria co-infections.^[53]^ Advocate for increased funding and support from governments, non-governmental organizations, and international bodies to address these co-infections effectively. Strengthen postnatal care services to continue monitoring and providing support to both mothers and infants, especially in regions with high prevalence of co-infections.^[53]^ Implement robust monitoring and evaluation systems to track the effectiveness of interventions, identify gaps, and guide evidence-based decision-making for program improvements. By focusing on these future directions and recommendations, healthcare systems and policymakers can work towards comprehensive strategies aimed at reducing the burden of HIV and Malaria co-infections in pregnant women, ultimately improving maternal and fetal health outcomes.

## 13. Conclusion

The intersection of maternal health with HIV and malaria presents multifaceted challenges that require comprehensive and integrated approaches to prevention, treatment, and care. Maternal and fetal outcomes are significantly influenced by factors such as disease severity, access to healthcare, and adherence to interventions. While maternal HIV and malaria infections can pose risks to both maternal and fetal health, effective strategies exist to mitigate these risks and improve outcomes. ART for HIV and IPTp for malaria are cornerstone interventions that have been shown to reduce vertical transmission rates and improve maternal and neonatal health outcomes. Additionally, health education, behavioral interventions, and community engagement play pivotal roles in promoting positive health behaviors, increasing awareness, and reducing stigma associated with HIV and malaria. Integrated maternal health services that address the dual burden of HIV and malaria are essential for optimizing outcomes and reducing maternal mortality and morbidity. By strengthening health systems, improving access to care, and addressing social determinants of health, countries can make significant strides towards achieving maternal and child health equity.

## Author contributions

**Conceptualization:** Emmanuel Ifeanyi Obeagu.

**Methodology:** Emmanuel Ifeanyi Obeagu, Getrude Uzoma Obeagu.

**Supervision:** Emmanuel Ifeanyi Obeagu.

**Validation:** Emmanuel Ifeanyi Obeagu.

**Visualization:** Emmanuel Ifeanyi Obeagu.

**Writing – original draft:** Emmanuel Ifeanyi Obeagu, Getrude Uzoma Obeagu.

**Writing – review & editing:** Emmanuel Ifeanyi Obeagu, Getrude Uzoma Obeagu.
